# Medical College and Hospital Notes

**Published:** 1897-12

**Authors:** 


### Medical College and Hospital Notes.

The cornerstone of the new building for the Bellevue Hospital Medical College, at First avenue and Twenty-sixth street, New York City, was laid with fitting ceremony Saturday afternoon, November 13, 1897, in the presence of a large audience. Most of the trustees and members of the faculty of the institution were present, and many alumni attended the ceremony, while others, including several women, were present by invitation. D. O. Mills, president of the Board of Trustees, presided at the ceremony. Among the other trustees present were Samuel Sloan, the Rev. Dr. Roderick Terry, Henry H. Porter and Thomas S. Brennan. Archbishop Corrigan, who is one of the trustees, sent a letter of regret.

Among the members of the faculty were Dr. Edward G. Janeway, president ; Dr. Austin Flint, secretary ; Dr. Lewis A. Sayre, emeritus professor of orthopedic and clinical surgery, and Drs. A. Alexander Smith, Hermann M. Biggs, Frederic S. Dennis, Joseph D. Bryant, Austin Flint, jr., George D. Stewart, Charles L. Dana, Henry C. Coe, William P. Northrup, Henry H. Rusby, Samuel Alexander, John A. Fordyce, Henry D. Noyes, Reginald H. Sayre, Carlos F. Macdonald, Beverly Robinson and Edward K. Dunham. Among others present were Bird S. Coler, Controller-elect ; Charities Commissioners Faure and O'Beirne, Sanitary Superintendent Roberts and Dr. John S. Billings, director of the New York Public Library.

The out-of-door ceremonies began at 3.30 o'clock, when a procession marched from the laboratory building to an awning that had been previously spread. An introductory address was delivered by Dr. Austin Flint, who, in conclusion, introduced Dr. Lewis A. Sayre, one of the founders of the college. Dr. Sayre, after a brief speech, proceeded to lay the cornerstone. At the conclusion of this ceremony the audience returned to the Carnegie Laboratory, where addresses were made by Dr. Landon Carter Gray, representing the alumni, the Rev. Dr. Terry, representing the trustees, and Dr. John S. Billings, who spoke for the medical profession. The Journal acknowledges an invitation to attend these ceremonies which it was compelled to regretfully decline.

---

The medical department of the University of Buffalo calls upon the profession of medicine in this city and vicinity to aid it in obtaining obstetric clinical material. Cases that cannot be well cared for at home may be sent to the Buffalo General Hospital, where two members of the senior classes will sleep at night and can be called to assist in cases where paid assistance cannot be afforded. The importance of obstetric clinics cannot be overestimated. Students should not be graduated who have not had ample obstetric experience. Physicians who can aid in supplying the material needed should communicate with Dr. Mann or Dr. Van Peyma.

